# The Use of Posturography in Vestibular Evaluation of Neurodegenerative Disorders: Diagnostic and Rehabilitative Impacts

**DOI:** 10.7759/cureus.88752

**Published:** 2025-07-25

**Authors:** Melissa Castillo-Bustamante, Cassandra Anderson, Veronica A Gutierrez

**Affiliations:** 1 Otolaryngology, Clinica Universitaria Bolivariana, Medellín, COL; 2 Otolaryngology, Universidad Pontificia Bolivariana, Medellín, COL; 3 Otolaryngology, School of Health Sciences, Universidad Pontificia Bolivariana, Medellín, COL; 4 Physiotherapy, M Health Fairview, Minneapolis, USA; 5 Physiotherapy, Interacoustics, Middelfart, DNK; 6 Audiology, Interacoustics Mexico, Mexico City, MEX

**Keywords:** alzheimer’s diseases, amyotrophic lateral sclerosis – frontotemporal spectrum disorder, multiple sclerosis, neurodegenerative disorders, parkinson' s disease, posturography, vestibular dysfunction

## Abstract

Neurodegenerative disorders, such as Parkinson's disease, multiple sclerosis, Alzheimer's disease, and amyotrophic lateral sclerosis, frequently present with vestibular dysfunction and balance disturbances, significantly impacting patients' quality of life and increasing the risk of falls. Vestibular impairments in these conditions can manifest as dizziness, unsteadiness, and difficulty maintaining postural control, further complicating the motor and cognitive deficits typical of these diseases. As a result, the assessment and management of balance dysfunction in neurodegenerative disorders have become crucial components of care. Posturography, an objective method for evaluating postural stability and balance control, has emerged as a valuable tool in the vestibular evaluation of patients with neurodegenerative conditions. By providing precise, quantitative measurements of balance deficits, posturography allows for a detailed analysis of postural control mechanisms that may be compromised due to vestibular involvement. This technique not only aids in diagnosing vestibular dysfunction but also plays a key role in developing targeted rehabilitation strategies. When integrated into vestibular rehabilitation (VR) programs, posturography can guide individualized therapy aimed at mitigating fall risk, improving functional mobility, and enhancing patients' overall quality of life. VR exercises, tailored to address specific balance deficits identified through posturographic analysis, can be particularly beneficial in promoting compensatory mechanisms and optimizing patients' functional abilities. This review highlights the significance of posturography as both a diagnostic and therapeutic tool in managing vestibular impairments associated with neurodegenerative diseases, offering the potential for improved outcomes through more personalized and effective rehabilitation interventions.

## Introduction and background

Neurodegenerative disorders are characterized by the progressive degeneration of the nervous system, often leading to widespread impairment of motor, cognitive, and sensory functions [1]. As these diseases progress, patients commonly experience balance difficulties, usually resulting from disruptions in the vestibular system [1,2]. The vestibular system is a key component of balance control, integrating sensory inputs from the inner ear, eyes, and proprioceptive systems to maintain equilibrium and spatial orientation [3]. When the vestibular system is compromised in neurodegenerative conditions such as Parkinson's disease (PD), multiple sclerosis (MS), and Alzheimer's disease (AD), patients frequently suffer from dizziness, unsteadiness, and an increased risk of falls [4]. These symptoms not only lead to physical injury but also significantly affect independence, mobility, and overall quality of life [4].

Given the complexity of vestibular dysfunction in these conditions, accurate and objective assessment methods are essential [4]. Posturography, a technique used to evaluate balance control by measuring the body’s center of pressure (CoP) during various static and dynamic tasks, has emerged as a valuable tool in this context [5]. Traditional clinical tests, such as the Romberg test or tandem walking, are useful but limited by their subjective nature and inability to provide detailed, quantitative data [6]. In contrast, posturography delivers a comprehensive, objective analysis of balance control, enabling clinicians to better diagnose vestibular impairments and assess the severity of balance disturbances in patients with neurodegenerative diseases [7].

In addition to its diagnostic capabilities, posturography plays a crucial role in vestibular rehabilitation (VR) [8]. By providing precise measurements of balance and postural control, posturography can guide individualized therapy, allowing for the targeted treatment of specific balance deficits [8]. Furthermore, it allows clinicians to monitor progress, adjusting rehabilitation programs as needed to maximize functional recovery [8].

Posturography, whether static or basic dynamic, is a technique used to evaluate postural sway by recording the CoP while a subject stands on a fixed or mildly unstable surface (such as a foam pad), typically with eyes open or closed [9]. It offers a general measure of balance control and is commonly used in simple clinical tests such as the tandem Romberg [10]. While it is accessible and low-cost, its diagnostic sensitivity is limited, as it does not allow differentiation between the visual, vestibular, and somatosensory contributions to balance [11]. Therefore, its utility is primarily in screening or identifying gross postural instability [12].

In contrast, computerized dynamic posturography (CDP) provides a more advanced and comprehensive assessment [13]. It involves a movable platform and visual surroundings that can be manipulated to challenge and isolate different sensory systems through standardized conditions, such as those used in the sensory organization test (SOT), which is a standardized component of computerized posturography, evaluates an individual’s ability to maintain balance under progressively altered sensory conditions by selectively disrupting visual, proprioceptive, and vestibular inputs across six testing scenarios, thus providing insight into how each sensory system contributes to postural control [13]. CDP allows for detailed analysis of how individuals integrate visual, vestibular, and proprioceptive inputs [13]. As highlighted by Visser et al., CDP is also valuable in documenting treatment effects, monitoring progression of disease, and improving understanding of postural control mechanisms [14].

This narrative review explores the current application of posturography in both the evaluation and rehabilitation of vestibular dysfunction in neurodegenerative diseases. It delves into the diagnostic value of this technology, its contribution to a deeper understanding of vestibular impairment mechanisms, and its integration into rehabilitation programs designed to improve balance, reduce fall risk, and enhance patients' quality of life.

## Review

Methods

Search Strategy

A narrative review of the literature was performed to identify relevant studies examining the use of posturography in the evaluation and rehabilitation of vestibular dysfunction in neurodegenerative diseases. The search was conducted using electronic databases, including PubMed, Scopus, and Google Scholar, covering publications from inception to 2024. The search strategy combined key terms such as “posturography,” “vestibular dysfunction,” “balance assessment,” “Parkinson’s disease,” “multiple sclerosis,” “Alzheimer’s disease”, “amyotrophic lateral sclerosis”, “neurodegenerative disorders,” and “vestibular rehabilitation.” Boolean operators (AND, OR) were employed to refine the search and capture relevant studies.

The inclusion criteria were established to focus on studies that directly addressed the role of posturography in neurodegenerative disorders and its use in vestibular rehabilitation. The inclusion criteria were peer-reviewed articles reporting on the application of posturography for assessing vestibular and balance dysfunction in patients diagnosed with neurodegenerative diseases, such as PD, MS, amyotrophic lateral sclerosis (ALS), and AD. Studies exploring the integration of posturography data into vestibular rehabilitation programs aimed at improving balance and reducing fall risk, research articles, clinical trials, and observational studies published in English, and studies involving adult human populations were included. Exclusion criteria included articles focusing on other neurological or non-neurodegenerative conditions, research on pediatric populations or animal models, conference abstracts, editorial letters, and non-peer-reviewed articles.

The extracted data included study design, population demographics, methods of posturography assessment, vestibular and balance outcome measures, and the role of posturography in the development of rehabilitation protocols. Discrepancies in data extraction were resolved through consensus. To ensure the reliability and validity of the included studies, a quality assessment was performed using criteria based on study design, sample size, methodology, and the clarity of posturography protocols. Studies were appraised for their use of appropriate controls, blinding methods (if applicable), and statistical rigor. The risk of bias was assessed for each study, and studies of low quality were excluded to enhance the scientific rigor of the review.

Following data extraction, a narrative synthesis was conducted to integrate findings from the included studies. This synthesis focused on identifying common themes related to the diagnostic accuracy of posturography in detecting vestibular dysfunction and its role in guiding vestibular rehabilitation interventions in neurodegenerative diseases. The findings were grouped by neurodegenerative condition, and the potential clinical applications of posturography in each context were highlighted. Gaps in the literature and areas for future research were also identified.

Neurodegenerative disorders and posturography challenges

Neurodegenerative disorders exhibit a broad spectrum of vestibular and postural control impairments due to the involvement of central nervous system pathways that coordinate balance. These impairments vary depending on the disease but all contribute significantly to mobility limitations and fall risk.

Parkinson’s Disease

PD is a neurodegenerative disorder characterized by the progressive degeneration of dopaminergic neurons in the substantia nigra, a key region of the basal nucleus, which is responsible for coordinating movement [15]. The cardinal motor symptoms of PD include bradykinesia (slowness of movement), muscular rigidity, resting tremors, and postural instability [16,17]. While motor deficits are well-documented, postural instability and balance dysfunction are among the most debilitating aspects of PD, significantly contributing to increased fall risk and reduced quality of life in affected individuals [16,17]. These balance disturbances are linked not only to motor impairments but also to vestibular dysfunction, a lesser recognized but important aspect of PD [16,17].

Vestibular dysfunction in PD arises from both peripheral and central mechanisms, impacting the ability to maintain balance and spatial orientation [18,19]. The vestibular system, which integrates inputs from the inner ear, brainstem, cerebellum, and cortical areas, plays a pivotal role in stabilizing vision during head movements and maintaining postural control [3]. In PD, abnormalities in vestibular-evoked myogenic potentials, inaccurate perception of head tilt, and visuospatial deficits have also been reported, further highlighting the contribution of vestibular and perceptual dysfunction to postural instability [16,19,20]. Vestibulo-ocular reflex (VOR) dysfunction, which destabilizes visual fixation during head movement, may contribute to dizziness and unsteadiness in PD patients [16]. More commonly, vestibular dysfunction in PD is attributed to central mechanisms [21]. The basal nucleus is integral in processing vestibular, proprioceptive, and visual inputs to maintain posture and balance [21,22]. Disruption in these circuits due to PD impairs sensory integration and postural control [21,22]. Furthermore, the progressive degeneration of brainstem nuclei involved in vestibular processing exacerbates balance deficits [22]. These central vestibular impairments are particularly evident in challenging conditions, such as walking on uneven surfaces or in low-light environments, where patients struggle to compensate for reduced or conflicting sensory input [21,22]. PD patients often exhibit impaired sensory integration, making it difficult to rely on vestibular information when visual or proprioceptive cues are compromised [16,21,22].

Posturography in PD 

Posturography is an important tool for evaluating postural instability and vestibular dysfunction in PD [12]. By quantitatively measuring body sway during various standing tasks, posturography provides objective data on postural control deficits that may not be visible through clinical observation alone [12]. In PD, this technology has proven particularly useful in identifying how patients rely on different sensory systems, visual, vestibular, and proprioceptive, for maintaining balance, and how the contributions of these systems evolve as the disease progresses [12]. The SOT, commonly employed in posturography, assesses a patient's ability to maintain balance when sensory inputs are manipulated [23]. The SOT measures postural sway across six conditions: standing with eyes open and closed on both firm and unstable surfaces, as well as standing with visual surroundings moving while on firm and unstable surfaces [23]. These conditions challenge a patient's ability to integrate vestibular, visual, and proprioceptive information for postural control [24]. In PD, studies using the SOT have consistently shown that patients rely heavily on visual input to maintain balance [22,24]. When visual input is removed, such as when standing with eyes closed, PD patients often exhibit increased sway and reduced postural stability [21,24]. This reliance on vision is thought to compensate for the impaired processing of vestibular and proprioceptive information, which are progressively affected by the disease [22,24].

Furthermore, patients with PD have difficulty adapting to changing sensory environments, which further compromises their balance [22,24]. As PD advances, posturography demonstrates a growing dependence on visual input, making patients particularly vulnerable to falls in low-light or visually complex environments [24,25]. SOT data also indicate a reduced ability to use vestibular information to stabilize posture when visual or proprioceptive cues are unavailable, underscoring the significant role of vestibular dysfunction in the postural instability observed in PD [24,25]. In PD, patients typically exhibit reduced limits of stability, reflecting their diminished ability to make controlled postural adjustments [24,25]. Posturography studies using LOS have revealed that patients with PD experience decreased dynamic postural control, particularly in advanced stages [24,25]. This reduced capacity for postural correction contributes to elevated fall risk and indicates a narrower margin of safety when performing everyday tasks such as reaching or bending [24,25]. Finally, postural sway analysis through posturography provides detailed insight into the small, involuntary movements the body makes to maintain balance while standing still [24,25]. In PD, postural sway is often exaggerated, particularly under challenging balance conditions, such as standing with eyes closed or on an unstable surface [24,25]. This suggests impaired integration of vestibular and proprioceptive information [20-25]. Posturography can also reveal abnormalities in sway direction and frequency, with PD patients frequently displaying more pronounced sway in the mediolateral (side-to-side) direction, which is associated with a heightened risk of falls [24,25]. 

Influence of Posturography on Diagnosis and Treatment of PD

The integration of posturography into the vestibular evaluation of PD has significantly enhanced both diagnostic accuracy and therapeutic approaches [24,25]. By providing objective, quantitative measurements of balance and postural control, posturography enables clinicians to detect vestibular dysfunction that may not be apparent through traditional clinical examinations [24,25]. Additionally, posturography facilitates the differentiation between vestibular and non-vestibular causes of postural instability, leading to more precise diagnoses [24,25]. One of the major challenges in PD management is the early detection of postural instability, which typically becomes more apparent in the later stages of the disease [26]. However, subtle balance impairments often manifest earlier in the disease course, and posturography is sensitive enough to identify these initial changes [26]. Early detection of vestibular dysfunction through posturography enables timely intervention, which may help delay the progression of postural instability and reduce the risk of falls [26]. Posturography is also instrumental in tailoring VR programs for PD patients [27]. In the context of PD, VR focuses on improving postural stability, dynamic balance, and the ability to adapt to changing sensory inputs [28]. By identifying the sensory systems that are most affected (vestibular, proprioceptive, or visual), posturography allows clinicians to customize VR exercises according to the patient's needs [28]. For example, if posturography reveals a heavy reliance on visual input, rehabilitation can emphasize enhancing proprioceptive and vestibular functions to reduce this dependence [16,28]. If the LOS test indicates reduced dynamic postural control, exercises can be tailored to improve the patient's ability to make controlled postural adjustments [28]. Several studies have demonstrated the efficacy of VR in improving balance and reducing the risk of falls in PD patients. Incorporating posturography into the VR process allows for ongoing monitoring of patient progress, enabling clinicians to make data-driven modifications to treatment plans [29]. This ensures that rehabilitation programs remain personalized and effective [29]. Posturography extends its utility beyond diagnosis and rehabilitation, playing a pivotal role in the long-term management of PD [29]. As postural instability typically worsens with disease progression, continuous monitoring of balance control is critical for adapting treatment strategies [29]. This device serves as a valuable tool for monitoring the progression of postural instability in PD over time [29]. Regular assessments provide clinicians with data on changes in balance performance, enabling timely adjustments to rehabilitation or medical interventions [29]. This is particularly beneficial in advanced stages of PD when postural instability becomes a leading cause of falls and mobility limitations [29].

Multiple Sclerosis

MS is a chronic inflammatory autoimmune disorder of the central nervous system (CNS) characterized by demyelination of nerve fibers and axonal degeneration [30]. This disease impacts various neurological functions depending on the location and extent of the lesions, with significant implications for balance and postural control [30]. The CNS plays a critical role in the integration of vestibular, proprioceptive, and visual information necessary for maintaining equilibrium. Consequently, vestibular dysfunction, dizziness, and postural instability are common symptoms experienced by patients with MS [30]. The increasing recognition of posturography as a vital tool for assessing balance impairments in MS patients has facilitated both diagnostic and rehabilitative strategies [30]. Vestibular dysfunction in MS often stems from the demyelination of neural pathways that process vestibular signals [31]. Lesions may affect any component of the vestibular system, including brainstem nuclei, cerebellar pathways, and the vestibulocochlear nerve (cranial nerve VIII) [31]. As a result, MS patients frequently report a range of vestibular symptoms, such as vertigo, imbalance, and dizziness [31]. These symptoms can severely compromise daily functioning and significantly elevate the risk of falls, exacerbated by sensory, motor, and coordination deficits inherent to the disease [31,32]. In MS, vestibular dysfunction can present in both central and peripheral forms, though central vestibular involvement is more prevalent due to the nature of the condition [25]. Central vestibular dysfunction may be characterized by abnormalities in the VOR, impaired sensory integration, and difficulties in maintaining postural control, particularly under challenging sensory conditions [33]. The heterogeneity of lesions throughout the CNS can lead to unique patterns of vestibular impairment among MS patients, making clinical evaluation complex [33]. In this context, posturography serves a crucial role by providing objective measures of balance control, enabling clinicians to assess the extent of balance dysfunction and the contributions of various sensory systems to postural stability [33].

Posturography in MS

Posturography is an essential tool for evaluating balance and vestibular dysfunction in patients with MS [34]. By quantifying parameters such as postural sway, limits of stability, and sensory integration during balance tasks, posturography provides valuable insights into the specific balance deficits experienced by individuals with MS [34]. This methodology enables the assessment of the contributions of different sensory systems, including visual, vestibular, and proprioceptive systems to postural control, as well as the impact of MS-related lesions on these systems [34]. The SOT is a widely utilized posturographic assessment designed to evaluate sensory integration and balance control in MS patients [35]. The SOT challenges individuals to maintain balance under six distinct conditions that progressively decrease reliance on visual, vestibular, and proprioceptive information [35]. Studies consistently demonstrate that MS patients exhibit difficulties in integrating sensory inputs, particularly when proprioceptive or visual information is either unreliable or absent [36]. Notably, posturographic evaluations using the SOT reveal that MS patients struggle to maintain balance in scenarios requiring vestibular input, such as standing with closed eyes or on unstable surfaces [36]. This reliance on visual cues to compensate for impaired vestibular function mirrors findings observed in other neurodegenerative conditions, such as Parkinson's disease [36]. However, the specific patterns of sensory reliance in MS may vary based on the location and extent of central nervous system lesions [36]. Additionally, MS patients often show increased postural sway in challenging sensory environments, indicating compromised vestibular compensation [36]. As the disease progresses, reliance on visual inputs tends to intensify, while the ability to effectively utilize vestibular and proprioceptive information for balance diminishes [36]. This decline is particularly pronounced in patients with cerebellar involvement, where ataxia and impaired postural control are prominent clinical features [36]. The limits of stability (LOS) test represents another critical posturographic measure that assesses dynamic balance control by evaluating how far an individual can lean in any direction without losing balance or stepping [37]. Patients with MS typically display reduced limits of stability, reflecting a compromised ability to execute controlled postural adjustments [38]. This impairment heightens fall risk due to the combined effects of motor weakness and poor coordination [38]. In studies utilizing the LOS test, MS patients frequently demonstrate diminished control over their center of mass and a reduced capacity for postural adjustments during voluntary movements [38]. Fatigue, a common symptom in MS, exacerbates these balance difficulties, further hindering the ability to maintain stability [38]. The LOS test yields crucial data for understanding the degree of postural instability in MS patients and can inform rehabilitation strategies aimed at enhancing dynamic balance [38]. Increased postural sway is frequently observed in MS, particularly when patients are challenged with difficult balance conditions, such as standing with their eyes closed or on unstable surfaces [39]. The sway patterns derived from posturography provide insights into the individual's capacity to utilize vestibular and proprioceptive inputs for balance maintenance [38]. Research indicates that MS patients often exhibit greater sway in both the anteroposterior (front-to-back) and mediolateral (side-to-side) directions, signifying compromised postural stability [39]. The magnitude of sway is generally correlated with the severity of the disease and the presence of cerebellar lesions, which are known to adversely affect coordination and balance [39]. Furthermore, posturography can detect subtle changes in sway over time, rendering it a valuable tool for monitoring disease progression and evaluating the effectiveness of rehabilitation interventions [39].

Influence of Posturography on Diagnosis and Treatment of MS

The application of posturography in the vestibular evaluation of MS has significantly enhanced the diagnosis of balance impairments and the development of tailored vestibular rehabilitation programs for affected patients [39]. By providing objective measures of postural control, posturography enables clinicians to identify vestibular dysfunction that may not be readily apparent through traditional clinical examination [39]. Furthermore, posturography facilitates the differentiation between balance deficits attributable to vestibular dysfunction and those resulting from motor or coordination impairments, thereby enabling more precise diagnoses and targeted therapeutic interventions [39]. In the context of MS, vestibular dysfunction and associated balance impairments can manifest early in the disease course, often before the onset of pronounced motor symptoms [30]. Early identification of these deficits is crucial for the timely implementation of interventions aimed at mitigating the progression of postural instability and reducing the risk of falls [30]. Posturography is particularly adept at detecting subtle changes in balance control, making it an optimal tool for identifying early vestibular dysfunction in MS patients [30]. By employing posturography to assess sensory integration and postural sway, clinicians can delineate specific patterns of vestibular impairment that may be indicative of CNS lesions affecting vestibular pathways [30]. For instance, an increased reliance on visual input for balance or impaired ability to utilize vestibular information in challenging sensory environments may suggest central vestibular dysfunction [30]. This early detection fosters more accurate diagnoses and provides critical insights for tailoring treatment strategies [30]. Posturography plays a pivotal role in guiding VR programs for MS patients [30]. VR, an exercise-based therapeutic approach, aims to improve balance and alleviate dizziness by promoting compensatory mechanisms for vestibular dysfunction [30]. In MS, VR programs typically concentrate on enhancing sensory integration, postural stability, and dynamic balance, all of which can be effectively assessed and monitored through posturographic evaluation [30]. By identifying the specific sensory systems that are compromised in MS patients (e.g., vestibular, proprioceptive, or visual), posturography empowers therapists to develop personalized rehabilitation programs that address the unique balance deficits of each patient [35]. For example, if posturography indicates that an MS patient excessively relies on visual input for balance, VR exercises may be designed to enhance proprioceptive and vestibular function to reduce this dependency [40]. Conversely, if posturography reveals inadequate dynamic postural control, rehabilitation efforts may emphasize exercises that challenge the patient’s limits of stability and promote effective postural adjustments during voluntary movements [40]. Posturography provides objective measures of balance and postural stability that can be tracked longitudinally to monitor disease progression in MS [40]. Regular posturographic assessments enable clinicians to detect subtle changes in balance performance that may indicate the deterioration of vestibular function or the emergence of new CNS lesions affecting balance control [40]. This ongoing monitoring is especially crucial for refining rehabilitation strategies and preventing falls as the disease evolves [36]. For example, if posturography reveals an increase in postural sway or a decline in sensory integration, it may signal that the patient’s balance is deteriorating, warranting more intensive rehabilitation interventions [40]. Additionally, posturography can aid in identifying when new vestibular rehabilitation strategies or assistive devices, such as balance aids, are necessary to maintain functional mobility and independence [40]. Falls represent a significant concern for individuals with MS, as they can lead to injuries, hospitalization, and a decline in quality of life [40,41]. By accurately identifying specific vestibular and balance deficits, posturography enhances the capacity to implement effective interventions aimed at reducing fall risk and improving the overall quality of life for MS patients [40,41].

Alzheimer’s Disease 

AD is primarily recognized as a cognitive disorder characterized by progressive neurodegeneration, notably marked by the accumulation of amyloid-beta plaques and tau neurofibrillary tangles [42]. While the cognitive decline associated with AD predominantly affects memory and executive function, there is an increasing body of evidence suggesting that vestibular dysfunction and balance impairments are also prevalent among individuals with this condition [43]. As the disease advances, damage to the hippocampus and other brain regions responsible for spatial orientation and balance can result in significant deficits in postural control. Consequently, patients with AD may experience symptoms such as dizziness, an increased frequency of falls, and challenges in navigating their environments [43]. The utilization of posturography in the evaluation of balance impairments in AD provides a quantitative assessment of postural instability and elucidates the interplay between cognitive decline and motor control [44]. Research indicates that individuals with AD exhibit a compromised ability to integrate sensory inputs from vestibular, visual, and proprioceptive systems, which contributes to heightened sway and balance deficits, particularly in dual-task scenarios that necessitate both cognitive and motor attention [44,45]. In addition to its well-documented effects on memory-related brain regions, Alzheimer's disease has been shown to impact other neurological systems critical for maintaining balance and processing vestibular information [45]. Vestibular dysfunction in AD may arise from both central and peripheral mechanisms, including degeneration of brainstem nuclei, cerebellar atrophy, and alterations within the vestibular apparatus itself [45]. Neuropathological changes in AD extend beyond the hippocampus to regions such as the parietal lobes and brainstem, both of which are integral to processing vestibular signals and integrating these signals with visual and proprioceptive information for balance maintenance [46]. Damage to these structures can impair the VOR, disrupt sensory integration, and compromise postural stability [4]. Empirical studies have demonstrated that individuals with AD exhibit abnormal VOR responses, indicating that central vestibular pathways are adversely affected by the neurodegenerative process [4]. Otopathologic findings have revealed degenerative changes in the vestibular end organs of patients, including a reduction in the number of hair cells within the semicircular canals and otolith organs [47,48]. This suggests that AD may exert a direct influence on the peripheral vestibular system, contributing to symptoms of dizziness, vertigo, and balance impairments [41,42]. Postural instability emerges as a prominent feature in AD, often manifesting even in the early stages of the disease [47-51]. As AD progresses, patients frequently demonstrate an increasing inability to maintain balance during routine activities, thereby elevating their risk of falls [51]. The underlying mechanisms of balance impairments in AD are multifactorial, encompassing both sensory processing deficits and motor dysfunction [49]. Patients with AD commonly experience challenges in integrating sensory inputs from the vestibular, visual, and proprioceptive systems, which are essential for effective postural control [50]. For instance, they may exhibit a reliance on visual input that becomes problematic in situations where visual information is unreliable, such as in low-light conditions or complex environments [50]. This phenomenon of sensory re-weighting can lead to diminished balance stability, particularly in challenging environmental contexts [50].

Posturography in AD

Posturography has emerged as a robust method for objectively assessing balance impairments in patients with AD [44]. This technique provides comprehensive information regarding postural sway, sensory integration, and dynamic balance control, which are crucial for elucidating the extent of vestibular dysfunction and balance instability characteristic of this condition [44]. The SOT is frequently employed to evaluate sensory integration in individuals with AD [51]. This test examines the patient’s ability to maintain balance across six distinct sensory conditions that manipulate the availability and reliability of visual, vestibular, and proprioceptive inputs [51]. Research utilizing the SOT in AD populations has consistently demonstrated that these patients encounter difficulties maintaining balance in conditions that particularly challenge vestibular function, such as standing on unstable surfaces or with eyes closed [51]. Impaired sensory integration in AD patients indicates a diminished capacity to compensate for unreliable or absent sensory information [52]. Furthermore, posturography studies have revealed that AD patients exhibit increased postural sway under challenging conditions, signifying inadequate vestibular compensation and an overreliance on visual input [53]. These findings underscore the critical role of vestibular rehabilitation in enhancing balance and mitigating fall risk within this demographic [52]. Individuals with AD typically demonstrate increased postural sway compared to healthy controls, particularly under challenging balance scenarios [53]. Empirical studies have indicated that AD patients exhibit greater sway in both the anteroposterior and mediolateral directions, which is indicative of compromised balance control [53]. These sway patterns tend to be exacerbated in conditions where vestibular or proprioceptive inputs are impaired, further emphasizing the contribution of vestibular dysfunction to balance impairments in AD [53]. Additionally, increased postural sway in AD patients has been correlated with both cognitive decline and the overall severity of the disease, suggesting that balance impairments deteriorate as the neurodegenerative process progresses [53]. The analysis of postural sway through posturography can detect subtle alterations in balance performance that may not be apparent through clinical observation alone, rendering it a valuable tool for the early identification of balance deficits in individuals with AD [53].

Influence of Posturography on Diagnosis and Treatment of AD

By providing objective metrics of balance performance and sensory integration, posturography enables clinicians to identify specific vestibular and balance deficits that may not be detectable through conventional clinical assessments [54]. Additionally, it has facilitated the development of targeted rehabilitative strategies aimed at improving balance and mitigating fall risk in patients with AD [54]. A primary advantage of posturography in AD is its capacity to detect early balance impairments, often prior to the emergence of significant motor symptoms [55,56]. Balance deficits related to AD can be subtle during the early stages of the disease, making detection via traditional clinical evaluations challenging [55]. However, posturography can reveal these early impairments by quantifying postural sway, sensory integration, and dynamic balance control under various challenging conditions [55]. For instance, patients with early-stage AD may exhibit increased postural sway or impaired sensory integration when visual or proprioceptive input is unreliable, indicating that vestibular function may already be compromised [49]. The ability to identify these early deficits through posturography allows clinicians to implement timely interventions that may help slow the progression of balance impairments and reduce the risk of falls [55]. Posturography has been instrumental in guiding VR programs tailored for AD patients [56]. Vestibular rehabilitation is an exercise-based therapeutic approach designed to enhance balance and reduce dizziness by promoting compensatory mechanisms for vestibular deficits [56]. In individuals with AD, VR programs typically target improvements in sensory integration, postural stability, and dynamic balance control, all of which can be evaluated and monitored through posturography [56,57]. By integrating posturography into the rehabilitation process, clinicians can track progress and make data-driven modifications to therapy programs, ensuring that patients receive the most effective interventions tailored to their specific balance impairments. Long-term management of balance impairments in AD is essential for sustaining functional independence and reducing fall risk [58]. As the disease progresses, balance impairments typically escalate, underscoring the importance of continuous assessment and intervention [58]. Posturography plays a crucial role in this ongoing management by providing objective measures of balance performance that can be longitudinally tracked [58]. Long-term management of balance impairments in AD is essential for sustaining functional independence and reducing fall risk [59]. As the disease progresses, balance impairments typically escalate, underscoring the importance of continuous assessment and intervention [59]. Posturography plays a crucial role in this ongoing management by providing objective measures of balance performance that can be longitudinally tracked [59]. Regular posturography assessments enable clinicians to monitor the progression of balance impairments in AD patients [55]. By consistently evaluating postural sway, sensory integration, and dynamic balance control, clinicians can detect subtle changes in balance performance that may indicate worsening vestibular dysfunction or the need for intensified rehabilitation [55]. This continuous monitoring is especially beneficial as AD advances, facilitating timely adjustments to treatment plans and fall prevention strategies [55]. For instance, an increase in postural sway or a decline in sensory integration identified through posturography may indicate a deterioration in the patient’s balance, suggesting the need for more intensive rehabilitation efforts [59]. Additionally, posturography can assist in determining the appropriateness of assistive devices, such as walkers or balance aids, to maintain functional mobility and independence [59]. Falls are a significant concern in AD patients, as they can result in injury, hospitalization, and a decline in quality of life [58,59]. By identifying specific vestibular and balance deficits through posturography, clinicians can implement targeted interventions aimed at reducing fall risk [60]. Effective strategies for improving balance and preventing falls in AD patients include vestibular rehabilitation, balance training, and the use of assistive devices. Moreover, posturography can inform the development of home-based fall prevention programs tailored to the patient’s unique balance impairments [59]. These programs may incorporate exercises designed to enhance sensory integration, strengthen postural muscles, and improve dynamic balance control [59]. By mitigating fall risk, these interventions can significantly enhance the quality of life for individuals with AD, enabling them to maintain greater independence for extended periods.

Amyotrophic Lateral Sclerosis

ALS is a progressive neurodegenerative disorder characterized by the degeneration of both upper and lower motor neurons, leading to progressive muscle weakness, spasticity, and ultimately respiratory failure [60]. While ALS is primarily recognized as a motor neuron disease, increasing evidence indicates that postural instability, vestibular dysfunction, and balance impairments are also prevalent among individuals with this condition [61]. These impairments substantially increase the risk of falls and contribute to reduced mobility and quality of life in ALS patients [60]. The mechanisms underlying balance disturbances in ALS are multifactorial, involving both motor and non-motor components. Progressive weakness of the trunk, neck, and lower limb muscles severely compromises the ability to maintain upright posture and perform postural adjustments [61-63]. Additionally, deficits in proprioceptive feedback and vestibular processing may further exacerbate instability [63]. Sanjak et al. demonstrated that vestibular deficits contribute to disequilibrium and falls in ambulatory ALS patients, highlighting the importance of including vestibular assessment in the clinical evaluation of this population [64]. Neuropathological studies suggest that degeneration extends beyond motor neurons in ALS, affecting structures involved in multisensory integration and balance control, such as the brainstem and cerebellum [64]. Krieg et al. reported that impaired trunk control is a key determinant of postural abnormalities in ALS, with trunk instability being strongly associated with abnormal posturographic findings [63]. These insights underscore the importance of using sensitive, quantitative tools such as posturography to capture the complex interplay of motor, vestibular, and sensory factors contributing to postural instability in ALS [63,64].

Posturography in ALS

Posturography offers a valuable means of objectively assessing balance impairments in ALS patients [63]. By quantifying postural sway, limits of stability, and sensory integration under various testing conditions, posturography provides detailed insights into the specific balance challenges faced by individuals with ALS [63]. Sanjak et al. used posturography to demonstrate that ALS patients exhibit significant deficits in sensory integration, particularly under conditions requiring increased reliance on vestibular input [64]. ALS patients often show increased postural sway when visual and proprioceptive cues are unreliable or absent, suggesting an impaired ability to compensate through vestibular pathways [64]. This phenomenon may be linked to both central processing deficits and progressive musculoskeletal deterioration, which together limit the capacity for effective postural corrections [64]. Similarly, Krieg et al. reported that ALS patients display marked abnormalities in trunk control during posturography tasks [63]. Increased mediolateral sway and reduced limits of stability are common findings, reflecting the progressive loss of dynamic balance control [60]. These posturographic abnormalities correlate with clinical measures of trunk weakness and functional disability, supporting the clinical relevance of incorporating posturography into ALS evaluation [63,64]. Furthermore, studies suggest that postural instability may emerge early in ALS, even before significant lower limb weakness occurs [64,65]. This highlights the potential utility of posturography for early detection of balance impairments and for monitoring disease progression over time.

Influence of Posturography on Diagnosis and Treatment of ALS

The integration of posturography into the clinical evaluation of ALS provides several key benefits. First, it enables the early identification of balance impairments that may not be readily apparent through routine clinical examination [63,64]. Given the high risk of falls in ALS, early detection of postural instability allows for the timely implementation of fall prevention strategies and targeted rehabilitation interventions [63,64]. Posturography also plays a crucial role in guiding rehabilitation programs for ALS patients. Although the progressive nature of ALS limits the potential for long-term improvement in motor function, VR and balance training can help optimize functional mobility and reduce fall risk [63,64]. By identifying specific sensory integration deficits, such as increased visual dependence or impaired vestibular compensation, posturography enables clinicians to tailor VR exercises to the individual needs of each patient [63,64]. Regular posturographic assessment provides objective data to monitor the progression of postural instability in ALS and to adjust rehabilitation strategies accordingly. For example, a decline in limits of stability or an increase in mediolateral sway may indicate the need for intensified therapy or the introduction of assistive devices to support safe mobility [63,64]. Importantly, posturography can also help assess the impact of rehabilitation interventions, providing evidence of their effectiveness and guiding ongoing clinical decision-making [63,64]. Finally, as ALS progresses and mobility becomes increasingly compromised, posturography can inform transitions to more supportive interventions, such as home modifications or powered mobility aids. By objectively characterizing balance impairments at each disease stage, posturography contributes to a comprehensive, proactive approach to managing postural instability in ALS [63,64].

Challenges and Considerations in Posturography Assessment

Neurodegenerative disorders like MS, PD, AD, and ALS manifest distinct yet often overlapping impairments in balance and postural control, impacting mobility and increasing fall risk among affected individuals [63-66]. Given the unique pathophysiological features of MS, PD, AD, and ALS, ranging from demyelination and neuroinflammation in MS, dopaminergic neuron loss in PD, widespread cortical degeneration in AD, to progressive motor neuron degeneration and trunk instability in ALS, posturography results can vary significantly between these populations. The complex interplay between sensory integration deficits, motor dysfunction, and cognitive decline across these conditions underscores the need for tailored posturography protocols that can sensitively capture disease-specific balance impairments and inform personalized rehabilitation strategies. [66]. Table 1 summarizes key posturography findings across MS, PD, AD, and ALS, highlighting the specific balance impairments associated with each disease and underscoring common challenges in sensory processing and motor control. These insights are crucial for developing tailored intervention strategies and enhancing the utility of posturography in clinical practice.

**Table 1:** Challenges and considerations in neurodegenerative disorders

	Challenges and considerations
Multiple Sclerosis	Disease variability: Multiple sclerosis (MS) patients show diverse progression, with some experiencing early balance issues and others remaining stable [63].
	Standardized posturography may miss subtle, disease-specific changes [64].
	Fatigue and cognitive impairment in MS: Fatigue and cognitive issues can disrupt posturography accuracy, often leading to variable and inconsistent balance results [64].
	Accessibility barriers: Mobility issues and lack of portable posturography tools limit access for MS patients, making assessments less reflective of real-world conditions [65].
	Optimizing assessment: Enhancing test sensitivity, accessibility, and portability are essential steps to improve balance evaluation for MS patients [65].
Alzheimer’s disease	Cognitive impairments affecting test reliability: Alzheimer's disease (AD) patients often have cognitive impairments, including memory and attention issues, which can hinder their ability to follow testing protocols and reduce the reliability of posturography results [50].
	Limitations of static testing: As balance issues worsen with AD progression, static posturography tests may fail to capture the dynamic balance challenges these patients face in everyday settings [51].
	Clinical vs. real-world discrepancies: Controlled clinical environments used for posturography may not reflect real-life situations, potentially underestimating balance difficulties that AD patients encounter in busy or visually complex spaces [50].
	Enhancing assessment validity: Improving ecological validity and incorporating dynamic balance assessments will be essential for accurately evaluating and addressing balance impairments in AD patients [51].
Parkinson’s Disease	Motor symptoms affecting test outcomes in Parkinson's disease (PD): motor symptoms like tremor, rigidity, and bradykinesia can interfere with posturography, leading to variable test results and potentially skewing assessments of postural control [22].
	Limited sensory integration assessment: PD patients often have impaired sensory integration, yet many standard posturography tests lack the sensitivity to detect subtle deficits in how visual, vestibular, and proprioceptive inputs are processed [22].
	The sensory organization test (SOT) provides a greater challenge to mediolateral postural control, which may lead to a better characterization of sensory contributions [23].
	Static testing vs. dynamic real-world needs: Standard posturography often emphasizes static balance, which may not reflect the dynamic postural adjustments PD patients need in real-world environments, where movement is constant and unpredictable [24].
	Bridging the gap in PD balance assessment: Enhancing posturography to assess dynamic and sensory integration aspects will improve balance evaluation accuracy for PD patients, leading to more effective interventions [22].
Amyotrophic Lateral Sclerosis	Progressive trunk weakness impairs upright balance control and can lead to abnormal mediolateral sway. Motor fatigue and respiratory dysfunction may limit endurance during posturography tasks [60].
	Cognitive and attentional impairments, though less common, can affect test performance in advanced amyotrophic lateral sclerosis (ALS) [61].
	Limited standardization of posturography protocols tailored to ALS-specific balance deficits hampers cross-study comparisons. Improved dynamic and trunk-focused assessment approaches are needed to better capture real-world postural challenges in ALS [60,61].

Future Directions for Enhancing Posturography Utility in Neurodegenerative Disorders

To address the challenges associated with posturography in PD, MS, AD, and ALS, several future directions warrant consideration. Customizing testing protocols for different stages of neurodegenerative diseases, such as early versus advanced stages of PD or AD, could enhance the sensitivity of balance assessments. For MS patients, employing shorter and more intuitive tasks may help mitigate the impact of cognitive fatigue, thus improving test performance [66]. Incorporating dynamic tasks into posturography assessments would provide a more comprehensive understanding of balance impairments across neurodegenerative disorders [66,67]. Developing posturography systems that assess balance control during dynamic activities such as gait initiation, turning, or obstacle avoidance could better capture real-life balance difficulties experienced by PD patients [66,67]. Wider access to posturography equipment and enhanced training for clinicians could expand the application of this technology in routine care. Developing lower-cost, portable posturography systems could improve access in regions with limited healthcare resources, particularly for MS, ALS, and AD patients residing in rural areas. Additionally, open-source software solutions could alleviate financial barriers to implementing posturography assessments in clinical practice. Increasing the availability of training programs for healthcare providers focused on interpreting posturography results and integrating them into treatment plans could enhance the utilization of this technology in managing balance impairments associated with neurodegenerative disorders. Current research has advanced the understanding of the vestibular system's involvement in neurodegenerative diseases such as PD, MS, AD, and ALS, yet significant potential remains for comprehensive vestibular testing to benefit these patients. Expanding research into vestibular function alongside tailored testing protocols could facilitate earlier diagnosis, improved disease management, and enhanced rehabilitation outcomes for individuals affected by these conditions.

## Conclusions

Posturography is a powerful tool for assessing balance and vestibular impairments in neurodegenerative disorders like PD, MS, ALS, and AD. However, several challenges, including cognitive and physical impairments, equipment constraints, and test sensitivity, limit its current utility. By adapting testing protocols, integrating real-world assessments, utilizing wearable technology, and improving access and training, posturography could become an even more effective tool in diagnosing, tracking, and treating balance impairments in these patient populations.

## References

[1] Lamptey RN, Chaulagain B, Trivedi R, Gothwal A, Layek B, Singh J (2022). A review of the common neurodegenerative disorders: current therapeutic approaches and the potential role of nanotherapeutics. Int J Mol Sci.

[2] Gao HM, Hong JS (2008). Why neurodegenerative diseases are progressive: uncontrolled inflammation drives disease progression. Trends Immunol.

[3] Yoo H, Mihaila DM (2025). Neuroanatomy, vestibular pathways. StatPearls [Internet].

[4] Cronin T, Arshad Q, Seemungal BM (2017). Vestibular deficits in neurodegenerative disorders: balance, dizziness, and spatial disorientation. Front Neurol.

[5] Sebastia-Amat S, Tortosa-Martínez J, Pueo B (2023). The use of the static posturography to assess balance performance in a Parkinson's disease population. Int J Environ Res Public Health.

[6] Ting KC, Lin YC, Chan CT, Tu TY, Shih CC, Liu KC, Tsao Y (2024). Inertial measurement unit-based Romberg test for assessing adults with vestibular hypofunction. IEEE J Transl Eng Health Med.

[7] Allum JH, Shepard NT (1999). An overview of the clinical use of dynamic posturography in the differential diagnosis of balance disorders. J Vestib Res.

[8] Keshner EA, Mallinson AI, Longridge NS, Sinno S, Petersen H, Perrin P (2022). Evolution of postural control assessment: from dynamic posturography to virtual reality. Front Neurol.

[9] Wiśniowska-Szurlej A, Ćwirlej-Sozańska A, Wilmowska-Pietruszyńska A, Sozański B (2022). The use of static posturography cut-off scores to identify the risk of falling in older adults. Int J Environ Res Public Health.

[10] Janc M, Sliwinska-Kowalska M, Politanski P, Kaminski M, Jozefowicz-Korczynska M, Zamyslowska-Szmytke E (2021). Posturography with head movements in the assessment of balance in chronic unilateral vestibular lesions. Sci Rep.

[11] Black FO (2001). What can posturography tell us about vestibular function?. Ann N Y Acad Sci.

[12] Badke MB, Miedaner JA, Shea TA, Grove CR, Pyle GM (2005). Effects of vestibular and balance rehabilitation on sensory organization and dizziness handicap. Ann Otol Rhinol Laryngol.

[13] Black FO (2001). Clinical status of computerized dynamic posturography in neurotology. Curr Opin Otolaryngol Head Neck Surg.

[14] Visser JE, Carpenter MG, van der Kooij H, Bloem BR (2008). The clinical utility of posturography. Clin Neurophysiol.

[15] Kouli A, Torsney KM, Kuan WL (2018). Parkinson’s disease: etiology, neuropathology, and pathogenesis. Parkinson’s Disease: Pathogenesis and Clinical Aspects.

[16] Petel A, Jacob D, Aubonnet R, Frismand S, Petersen H, Gargiulo P, Perrin P (2022). Motion sickness susceptibility and visually induced motion sickness as diagnostic signs in Parkinson's disease. Eur J Transl Myol.

[17] Magrinelli F, Picelli A, Tocco P (2016). Pathophysiology of motor dysfunction in Parkinson's disease as the rationale for drug treatment and rehabilitation. Parkinsons Dis.

[18] Gui M, Lv L, Qin L, Wang C (2024). Vestibular dysfunction in Parkinson's disease: a neglected topic. Front Neurol.

[19] Smith PF (2018). Vestibular functions and Parkinson's disease. Front Neurol.

[20] Palakurthi B, Burugupally SP (2019). Postural instability in Parkinson's disease: a review. Brain Sci.

[21] Wagner AR, Merfeld DM (2023). A modified two-dimensional sensory organization test that assesses both anteroposterior and mediolateral postural control. Front Rehabil Sci.

[22] Barbosa AF, Chen J, Freitag F, Valente D, Souza CO, Voos MC, Chien HF (2016). Gait, posture and cognition in Parkinson's disease. Dement Neuropsychol.

[23] Rossi-Izquierdo M, Basta D, Rubio-Rodríguez JP (2014). Is posturography able to identify fallers in patients with Parkinson's disease?. Gait Posture.

[24] Nieto-Escamez F, Obrero-Gaitán E, Cortés-Pérez I (2023). Visual dysfunction in Parkinson's disease. Brain Sci.

[25] Becker D, Maric A, Schreiner SJ, Büchele F, Baumann CR, Waldvogel D (2022). Onset of postural instability in Parkinson's disease depends on age rather than disease duration. Parkinsons Dis.

[26] Soto-Varela A, Rossi-Izquierdo M, Del-Río-Valeiras M, Vaamonde-Sánchez-Andrade I, Faraldo-García A, Lirola-Delgado A, Santos-Pérez S (2020). Vestibular rehabilitation using posturographic system in elderly patients with postural instability: can the number of sessions be reduced?. Clin Interv Aging.

[27] Feng H, Li C, Liu J (2019). Virtual reality rehabilitation versus conventional physical therapy for improving balance and gait in Parkinson's disease patients: a randomized controlled trial. Med Sci Monit.

[28] Kwon SH, Park JK, Koh YH (2023). A systematic review and meta-analysis on the effect of virtual reality-based rehabilitation for people with Parkinson's disease. J Neuroeng Rehabil.

[29] Papiri G, D'Andreamatteo G, Cacchiò G (2023). Multiple sclerosis: inflammatory and neuroglial aspects. Curr Issues Mol Biol.

[30] Pula JH, Newman-Toker DE, Kattah JC (2013). Multiple sclerosis as a cause of the acute vestibular syndrome. J Neurol.

[31] Smith LJ, Wilkinson D, Bodani M, Surenthiran SS (2024). Cognition in vestibular disorders: state of the field, challenges, and priorities for the future. Front Neurol.

[32] Grassi L, Rossi S, Studer V (2017). Quantification of postural stability in minimally disabled multiple sclerosis patients by means of dynamic posturography: an observational study. J Neuroeng Rehabil.

[33] Mulavara AP, Cohen HS, Peters BT, Sangi-Haghpeykar H, Bloomberg JJ (2013). New analyses of the sensory organization test compared to the clinical test of sensory integration and balance in patients with benign paroxysmal positional vertigo. Laryngoscope.

[34] Fling BW, Dutta GG, Schlueter H, Cameron MH, Horak FB (2014). Associations between proprioceptive neural pathway structural connectivity and balance in people with multiple sclerosis. Front Hum Neurosci.

[35] Podda J, Marchesi G, Bellosta A (2024). Testing dynamic balance in people with multiple sclerosis: a correlational study between standard posturography and robotic-assistive device. Sensors (Basel).

[36] Clark S, Rose DJ, Fujimoto K (1997). Generalizability of the limits of stability test in the evaluation of dynamic balance among older adults. Arch Phys Med Rehabil.

[37] Cameron MH, Lord S (2010). Postural control in multiple sclerosis: implications for fall prevention. Curr Neurol Neurosci Rep.

[38] Prosperini L, Castelli L (2018). Spotlight on postural control in patients with multiple sclerosis. Degener Neurol Neuromuscul Dis.

[39] Warmerdam E, Schumacher M, Beyer T (2022). Postural sway in Parkinson's disease and multiple sclerosis patients during tasks with different complexity. Front Neurol.

[40] Hebert JR, Corboy JR, Manago MM, Schenkman M (2011). Effects of vestibular rehabilitation on multiple sclerosis-related fatigue and upright postural control: a randomized controlled trial. Phys Ther.

[41] Fritz NE, Newsome SD, Eloyan A, Marasigan RE, Calabresi PA, Zackowski KM (2015). Longitudinal relationships among posturography and gait measures in multiple sclerosis. Neurology.

[42] Kumar A, Sidhu J, Lui F, Tsao JW (2025). Alzheimer disease. StatPearls [Internet].

[43] Agrawal Y, Smith PF, Rosenberg PB (2020). Vestibular impairment, cognitive decline and Alzheimer's disease: balancing the evidence. Aging Ment Health.

[44] Sant'Anna P, Silva FO, Rodrigues AC (2019). Posturographic analysis of older adults without dementia and patients with Alzheimer's disease: a cross-sectional study. Dement Neuropsychol.

[45] Lee SC (2017). Relationship of visual dependence to age, balance, attention, and vertigo. J Phys Ther Sci.

[46] Aedo-Sanchez C, Riquelme-Contreras P, Henríquez F, Aguilar-Vidal E (2024). Vestibular dysfunction and its association with cognitive impairment and dementia. Front Neurosci.

[47] Mirino P, Pecchinenda A, Boccia M, Capirchio A, D'Antonio F, Guariglia C (2022). Cerebellum-cortical interaction in spatial navigation and its alteration in dementias. Brain Sci.

[48] Kobel MJ, Wagner AR, Merfeld DM, Mattingly JK (2021). Vestibular thresholds: a review of advances and challenges in clinical applications. Front Neurol.

[49] Agrawal Y, Zuniga MG, Davalos-Bichara M, Schubert MC, Walston JD, Hughes J, Carey JP (2012). Decline in semicircular canal and otolith function with age. Otol Neurotol.

[50] Albers MW, Gilmore GC, Kaye J (2015). At the interface of sensory and motor dysfunctions and Alzheimer's disease. Alzheimers Dement.

[51] Fujita K, Sugimoto T, Noma H (2024). Postural control characteristics in Alzheimer's disease, dementia with Lewy bodies, and vascular dementia. J Gerontol A Biol Sci Med Sci.

[52] Grove CR, Whitney SL, Hetzel SJ, Heiderscheit BC, Pyle GM (2021). Validation of a next-generation sensory organization test in adults with and without vestibular dysfunction. J Vestib Res.

[53] Zhang NK, Zhang SK, Zhang LI, Tao HW, Zhang GW (2023). Sensory processing deficits and related cortical pathological changes in Alzheimer's disease. Front Aging Neurosci.

[54] Bollinger RM, Chen SW, Krauss MJ (2024). The association between postural sway and preclinical Alzheimer disease among community-dwelling older adults. J Gerontol A Biol Sci Med Sci.

[55] Mirka A, Black FO (1990). Clinical application of dynamic posturography for evaluating sensory integration and vestibular dysfunction. Neurol Clin.

[56] Buckley C, Alcock L, McArdle R (2019). The role of movement analysis in diagnosing and monitoring neurodegenerative conditions: insights from gait and postural control. Brain Sci.

[57] Gago MF, Yelshyna D, Bicho E, Silva HD, Rocha L, Lurdes Rodrigues M, Sousa N (2016). Compensatory postural adjustments in an oculus virtual reality environment and the risk of falling in Alzheimer's disease. Dement Geriatr Cogn Dis Extra.

[58] Catania V, Rundo F, Panerai S, Ferri R (2023). Virtual reality for the rehabilitation of acquired cognitive disorders: a narrative review. Bioengineering (Basel).

[59] Bollinger RM, Keleman A, Thompson R (2021). Falls: a marker of preclinical Alzheimer disease: a cohort study protocol. BMJ Open.

[60] González-Sánchez M, Ramírez-Expósito MJ, Martínez-Martos JM (2025). Pathophysiology, clinical heterogeneity, and therapeutic advances in amyotrophic lateral sclerosis: a comprehensive review of molecular mechanisms, diagnostic challenges, and multidisciplinary management strategies. Life (Basel).

[61] Liu X, Chen L, Ye S, Liu X, Zhang Y, Fan D (2024). Postural instability and lower extremity dysfunction in upper motor neuron-dominant amyotrophic lateral sclerosis. Front Neurol.

[62] Brotman RG, Moreno-Escobar MC, Joseph J, Munakomi S, Pawar G (2025). Amyotrophic lateral sclerosis. StatPearls [Internet].

[63] Krieg I, Dalin D, Heimbach B, Wiesmeier IK, Maurer C (2019). Abnormal trunk control determines postural abnormalities in amyotrophic lateral sclerosis. NeuroRehabilitation.

[64] Sanjak M, Hirsch MA, Bravver EK, Bockenek WL, Norton HJ, Brooks BR (2014). Vestibular deficits leading to disequilibrium and falls in ambulatory amyotrophic lateral sclerosis. Arch Phys Med Rehabil.

[65] Yesantharao LV, Rosenberg P, Oh E, Leoutsakos J, Munro CA, Agrawal Y (2022). Vestibular therapy to reduce falls in people with Alzheimer's disease: study protocol for a pilot randomized controlled trial. Pilot Feasibility Stud.

[66] Rinalduzzi S, Trompetto C, Marinelli L (2015). Balance dysfunction in Parkinson's disease. Biomed Res Int.

[67] Minta K, Colombo G, Taylor WR, Schinazi VR (2023). Differences in fall-related characteristics across cognitive disorders. Front Aging Neurosci.

